# Supplementary material for: Are carbon emissions trading and green financial instruments synergistic? -Comprehensive quantitative research based on content analysis
Source: PLoS One. 2024 Mar 7;19(3):e0298601. doi: 10.1371/journal.pone.0298601 (PMC10919601; doi:10.1371/journal.pone.0298601)
Supplement: S1 Data — (ZIP) [file pone.0298601.s001.zip › Data/地级市Z_F工作报告.zip!/╕ú╜¿/┴·╤╥╩╨/└┤╫╘╣1⁄2╓┌║┼í╛┬φ┐╦╩2╛▌═°í┐_2008.docx]

——2008年3月26日在龙岩市第三届人民代表大会第三次会议上

各位代表：

现在，我代表市人民政府向大会作政府工作报告，请予审议，并请市政协委员和各位列席人员提出意见。

一、2007年工作回顾

2007年，是本届政府任期的第一年。一年来，我市各级政府在省委、省政府和市委的正确领导下，认真学习贯彻党的十六大、十七大精神，坚持以邓小平理论和“三个代表”重要思想为指导，深入贯彻落实科学发展观，积极融入海峡西岸经济区建设，加快建设生态型经济枢纽和海西重要增长极，全市经济社会发展呈现增长较快、结构优化、效益提高、民生改善的良好态势。初步统计，全市实现地区生产总值556亿元，增长16%，实现1995年以来的最快增长，增速居全省第四位，总量提升到全省第五位；人均地区生产总值20182元，增长15.6%；全社会固定资产投资248.6亿元，增长50.8%；全部工业总产值639.5亿元，增长30.6%；财政总收入96.5亿元，增长26.1%，其中地方级财政收入36.7亿元，增长28.7%；社会消费品零售总额167.7亿元，增长15%；居民消费价格总水平上涨5.9%；城镇居民人均可支配收入14128元、实际增长10.7%，农民人均纯收入5086元、实际增长6.9%。

1、扎实推进新农村建设，农业和农村面貌出现可喜变化。支农惠农力度加大，各级投入支农资金6.3亿元，增长93.6%。农林牧渔业总产值179.8亿元，增长6.1%。粮食总产稳中略增，烤烟、水果、蔬菜生产平稳发展，竹业、茶业、食用菌加快发展；完成植树造林33万亩；生猪出栏602万头，规模化生态养殖力度加大。动植物疫情疫病防控继续加强。森华公司年加工100万头生猪冷鲜肉生产线、长汀盼盼休闲食品加工生产线等一批农产品加工项目建成投产，农业产业化经营稳步推进，农民专业合作组织加快发展。漳平台湾农民创业园建设取得重大进展。完成农村劳动力技能培训5.8万人。农村“六通”工程建设进展顺利，实现村村通电、通电话、通饮用水、通广播电视、通水泥道路，68%的村通客车；“六千”水利工程超额完成年度任务，实施土地整理16.1万亩，改造中低产田5.9万亩，新建户用沼气池1万口；防灾减灾救灾体系不断健全，防汛防台风预警应急体系建设经验在全省推广。新建182个村级组织活动场所并投入使用，“万村千乡”市场工程、农村现代化流通网络工程建设扎实推进。农村“家园清洁行动”取得积极成效。村庄规划和小城镇建设得到加强。第二次全国农业普查工作任务顺利完成。

2、着力壮大重点产业，新型工业化进程不断加快。“10+3”产业中的11个工业产业完成规模以上工业总产值501.7亿元，增长39.4%，其中机械产业产值突破80亿元，烟草产业产值突破70亿元，机械、建材、钢铁、铜、农产品加工、电子信息6个产业产值增幅超过40%。全市净增规模以上工业企业167家、累计976家，净增产值亿元以上工业企业40家、累计100家；8个省级工业园区规模以上工业产值增长58.4%，15个乡镇工业集中区建设取得新进展；实现规模以上工业总产值538亿元，增长35.4%。工业对GDP增长的贡献率达58.9%，同比提高0.8个百分点。新增国家级高新技术企业1家、累计4家，省级高新技术企业11家、累计26家，省级企业技术中心2家、累计6家。新增中国驰名商标、省著名商标和中国、省名牌产品以及国家免检产品20个，累计103个。新罗区被亚洲制造业协会评为“2007中国制造业最具发展潜力城市”。关闭水泥污染企业24家、机立窑生产线62条，淘汰落后水泥产能655万吨。规模以上工业经济效益综合指数从2004年4月起连续45个月居全省首位，万元规模工业增加值能耗下降0.4%，二氧化硫排放量下降9%，化学需氧量减少2.4%。服务业加快发展，第三产业增加值增长14%。实现旅游总收入50.2亿元，增长10%。交通运输、金融、商贸、物流、房地产和社区服务业较快发展。

3、深入实施项目带动，项目工作取得显著成效。“三个一百”项目中，193个新增长点项目新增产值118.8亿元，增长45.6%；288个投资项目完成投资173.9亿元，增长51.3%；163个储备项目中29个项目建成投产或开工建设。110个重点项目完成投资123.1亿元，增长58%。全市完成工业投资96.8亿元，增长26.6%。龙工齿轮和变速箱加工、长汀凯鲍专用汽车生产线、漳平红狮一期新型干法水泥等项目建成投产；紫金山金铜矿联合开采、武平塔牌新型干法水泥、永定畅丰汽车前轴和工程机械铸锻件加工、连城正鑫精密机械制造等项目加快建设；龙烟精品“七匹狼”卷烟生产线技改、龙工工程机械液压设备、龙工工程机械铸锻件加工、希尔斯高档电梯、紫金恒发工业园等项目开工建设；上杭紫金年产20万吨铜冶炼、马坑铁矿新增500万吨/年采选工程等项目前期工作顺利推进。参加第五届“6·18”项目成果交易会和第十一届“9·8”投洽会等取得积极成果，成功举办建市十周年系列活动和第四届“11·18”中国龙岩投资项目洽谈会，促成了一大批项目签约、开工和竣工。“双百”项目竞赛成效明显，全市总投资3000万元以上的生产性项目新签约437个、新开工365个、新竣工投产231个。

4、加强基础设施建设，投资环境日益优化。冠豸山机场新开通龙岩—上海航班，龙长高速公路、古田大道建成通车，永武高速公路、龙厦铁路全线开工。新增高速公路里程136公里、累计174公里，基本完成国道和通县道路路面改造，完成农村公路路面硬化1900公里、累计7600公里。建成22万伏和11万伏输变电工程7个、累计40个。永春至永定高速公路、赣龙铁路复线、龙岩50万伏输变电工程和坑口火电厂二期、漳平电厂改扩建项目前期工作取得实质性进展。机关效能建设和政府系统绩效评估工作取得新成绩。全市新获批工业用地1.5万亩，增长36.4%；金融机构人民币贷款余额343.4亿元，增长27.5%；向“10+3”产业新输送技术工人和熟练工人2.1万人。完成部门内部相对集中行政审批职能改革，新增永定、漳平两个县级行政服务中心，办事效率和服务水平进一步提升。大通关环境不断改善。

5、强化中心城市规划建设管理，集聚带动能力逐步提升。进一步推进中心城市总体规划修编工作，规划对城市建设的引导和调控作用进一步增强。启动了中心城市商务、物流商贸和人居三大板块的规划建设。2003—2007年滚动实施的百个城建项目基本完成，累计投资74.9亿元，是计划的1.2倍。城市道路增加15公里、累计300公里，城市污水集中处理率80.1%，生活垃圾无害化处理率95.4%，建成区绿地覆盖率36.6%，建成区面积增加1.7平方公里、扩大到33.3平方公里。第二水源项目建设稳步推进。完成中心城市第四期夜景灯光工程建设。龙岩经济开发区和龙州工业园区新落户工业企业80家、累计294家，新增3家四星级旅游饭店，城市公交事业稳步发展。加强城市管理执法，坚决清理整治各类违法违章建设，拆除中心城市违法违章建设10.7万平方米，市容环境明显改善。

6、深化改革开放，发展动力不断增强。农村改革有序推进，在全省率先实施并完成市、县、乡三级兽医管理体制改革，100%的村完成集体林权制度改革，生态公益林管护机制改革全面启动，建成19个乡镇“三农”服务中心和226个“农民之家”，乡镇机构改革试点、农村金融改革取得新进展。国有企业改革继续深化，完成19家市属国有企业职工身份置换，累计身份置换面达100%。新增股份公司4家，10家企业进入上市辅导期。国有资产监管得到加强，处置不良资产力度加大。市对龙岩经济开发区和新罗区财政管理体制平稳调整到位，政府收支分类改革、国库集中支付试点工作稳步推进。事业单位分类改革和人事制度、投资体制、社会事业等领域改革迈出新步伐。全市实际利用外资3.7亿美元，增长54%；内联引资实际利用市外资金74.8亿元，增长55.8%；外贸出口2.4亿美元，增长56.5%；登记注册资本1000万元以上的私营企业539户，增长36.8%。龙台经贸稳步发展，交流合作不断加强。与厦门的山海协作、厦泉漳龙城市联盟和闽西南五市、闽粤赣十三市区域协作取得新成效。

7、加快发展社会事业，人民群众得到更多实惠。组织开展了首届科学技术突出贡献奖评选活动，认真实施各类科技重点计划、产学研合作项目，27个项目被列入国家、省科技项目计划，获专利189件、增长69%，实现高新技术产业产值90亿元、增长49%，市科技创业园建设稳步推进。教育事业健康发展，义务教育均衡发展水平稳步提高，新罗区、永定县通过省政府“双高普九”评估验收，高等教育和职业教育发展规模与质量不断提升，普通高考万人口上本科线人数连续6年居全省第一。继续对农村义务教育阶段学生实行“两免一补”政策，启动城市义务教育阶段低保家庭学生免除学杂费、免费提供教科书工作，第三期28.8万平方米农村中小学D级危房改造全面推进，农民工子女入学得到有效保障。永定土楼申报世界文化遗产通过国际古迹遗址理事会专家的考察评估，启动了冠豸山申报世界自然遗产工作，完成古田会议会址群维修保护二期工程建设，古田镇被列入国家级历史文化名镇，闽西革命历史博物馆改版、革命传统教育基地维修保护、乡镇文化站建设顺利推进，成功举办纪念胡文虎先生诞辰125周年活动。新型农村合作医疗覆盖全市、农民参合率达85%，17所县级以上医院对口帮扶125所乡镇卫生院，从县级以上医院选派了百名医生帮扶百所乡镇卫生院，乡镇卫生院“百院建设、千人培训”工作成效明显，公共卫生服务、医疗服务和医疗保障体系不断健全，城乡卫生保健水平不断提高。卫生、食品和药品监督管理力度加大，产品质量和食品安全专项整治取得阶段性成果。连续17年完成省下达人口控制计划。全民健身广泛开展，竞技体育取得新成绩。有线数字电视整体转换工作全面启动，新增606个20户以上自然村通广播电视。新增城镇就业2.7万人，城镇登记失业率3.95%、同比下降0.75个百分点。社会保险覆盖面不断扩大，农村居民最低生活保障标准从每年1000元提高到1200元，60.6万户农民房屋全部纳入统一保险，实施了部分离任村（居）主干养老补助，在全省率先实施村妇代会主任生活津贴和符合条件的离任村妇代会主任养老补助；社会救助、社会福利、优抚安置工作得到加强，市福利中心开工建设，慈善事业取得新发展；水库移民后期扶持政策全面落实，实施“造福工程”搬迁994户、4601人。新建经济适用房7.6万平方米，廉租房1.36万平方米。封山育林和“青山挂白”、水土流失治理工作持续加强，汀江、九龙江、闽江流域水环境综合整治稳步推进，连续8年实现耕地占补平衡，中心城市　　环境空气质量优良以上天数达305天、同比增加18天。安全生产“四项指数”连续3年全面下降。市委、市政府确定的25项为民办实事项目得到全面落实。“双拥”共建、国防动员、国防教育、人民武装和人民防空工作取得新成绩，龙岩市、连城县获得新一届全国“双拥”模范城（县）称号。人事、机构编制、统计、民族宗教、外事侨务、老区、库区移民、对口援藏、信访、气象、地震、档案、地方志、科普、老龄、妇女儿童、残疾人等工作取得新进展。

8、坚持依法行政，民主法制建设取得新进步。认真贯彻《监督法》，依法接受人大及其常委会的法律监督和工作监督，认真执行人大及其常委会的决议决定，积极配合人大常委会开展执法检查、人大代表视察和专项调研。支持政协履行政治协商、民主监督和参政议政职能，加强与各民主党派、无党派人士、工商联和人民团体的联系，虚心听取社会各界人士的建议意见。市三届人大一次、二次会议和市政协三届一次会议以来，交由政府系统承办的277件市人大代表建议和484件市政协提案已全部办结。“五五”普法深入开展，行政执法行为进一步规范，廉政建设、政务公开、行政监察、审计监督等工作不断加强。完善了突发公共事件总体应急预案和专项预案，处理突发公共事件、保障公共安全能力有效增强。创建“平安龙岩”取得明显成效，居民对社会治安满意率达96.4%、同比提高2个百分点，我市成为全省唯一全部县（市、区）通过平安验收的设区市。社区建设稳步推进。创建省级文明城市工作持续加强，群众性精神文明创建活动深入开展。

各位代表，过去一年我们所取得的成绩来之不易，这是在省委、省政府和市委的正确领导下，在市人大及其常委会的法律监督、工作监督和市政协的民主监督下，全市人民齐心协力、艰苦奋斗和社会各界大力支持的结果。在此，我代表市人民政府，向为经济社会发展付出辛勤劳动的全市人民，向给予政府工作大力支持与有效监督的人大代表和政协委员、各民主党派、无党派人士、工商联、各人民团体、离退休老同志和社会各界人士，向为龙岩改革发展稳定作出积极贡献的中央和省直各部门及驻岩机构、驻岩部队、武警官兵、公安政法干警，向所有关心支持龙岩发展的港澳台同胞、海外侨胞、国际友人，表示衷心的感谢和崇高的敬意！

在肯定成绩的同时，我们也清醒地看到，当前龙岩经济社会发展还面临不少困难和问题，主要是：经济总量偏小，与沿海发达地区的差距仍然较大；先进制造业、服务业发展相对滞后，能够有效支撑带动重点产业发展的大项目比较缺乏；人口、资源、环境压力较大，节能减排工作任务重；城乡、区域发展不平衡，乡村债务较重，农业基础比较薄弱，农民持续增收难度加大；劳动就业、社会保障、教育、医疗卫生、住房、安全生产、社会治安等领域需要解决的问题还比较多，主要食品和居住类价格上涨幅度偏高，低收入群众生活比较困难；政府职能转变还不到位，一些政府机关工作人员服务意识不强、素质不高，形式主义、官僚主义和消极腐败现象仍然存在，机关作风建设有待加强。对这些问题，我们将认真对待，采取有力措施，认真加以解决。

二、2008年工作安排

各位代表，2008年是全面贯彻党的十七大精神的第一年，也是实施“十一五”规划、推进又好又快发展、建设生态型经济枢纽和海西重要增长极的关键一年。政府工作的总体要求是：全面贯彻党的十七大精神，高举中国特色社会主义伟大旗帜，以邓小平理论和“三个代表”重要思想为指导，深入贯彻落实科学发展观，积极融入海西“两个先行区”建设，围绕“四谋发展”实践主题，坚持“四个重在”实践要领，落实“四个关键”实践要求，朝着“四求先行”实践方向，按照市第三次党代会和市委三届四次全会的部署，更加重视推进改革开放和自主创新，更加重视转变经济发展方式和提高发展质量，更加重视节约资源和保护环境，深化项目带动，实施品牌带动，推进产业、基础设施、城市三大体系和新农村建设，着力民生民心，促进社会和谐，发挥海西纵深推进的前锋作用，加快把龙岩建设成为生态型经济枢纽和海西重要增长极。

经济社会发展的主要预期目标是：地区生产总值增长12.5%；财政总收入、地方级财政收入均增长13%；全社会固定资产投资增长21%；实际利用外资增长20%，内联引资实际利用市外资金增长30%，外贸出口增长20%；城镇登记失业率控制在4%以内；社会消费品零售总额增长13%，居民消费价格总水平涨幅略低于上年；人口自然增长率控制在9‰以内；城镇居民人均可支配收入增长10%，农民人均纯收入增长5.5%；单位生产总值能耗降低4.4%，二氧化硫排放量下降1.1%，化学需氧量减少0.05%。各项指标在执行中，力争完成得更好更快些。

为实现上述目标，市政府将着力做好以下工作：

1、坚持稳中求进，着力保持经济平稳较快发展。抓住国家继续支持东部地区率先发展、支持海峡西岸经济发展、加大对革命老区发展扶持力度的机遇，精心谋划，有效运作，持续经济平稳较快发展的良好势头。

积极推进海峡西岸产业集中区建设。围绕做大做强“10+3”产业，进一步加强领导、细化目标、统筹协调、改进服务，集中优势资源和力量，确保机械产业年产值率先突破100亿元，其他产业加速发展。组织开展工业发展竞赛活动，继续实施工业“百家千户”培育工程，力争净增规模以上工业企业120家、产值亿元以上工业企业25家。大力发展园区经济，进一步完善8个省级工业园区和15个乡镇工业集中区的基础设施和配套设施，强化服务功能，促进项目组团和产业集聚。加强对服务业发展的领导，研究制订促进服务业发展和扶持服务业重点项目建设的政策措施，在大力发展物流、商务、交通运输、金融、信息服务等生产性服务业的同时，加快发展旅游、商贸、房地产、社区服务等消费性服务业。加强对经济运行的监测预警，引导金融机构加大对地方经济发展的投入，办好第四届银企洽谈会，促进中小企业信用担保体系建设，保障项目用地，加强煤电油运协调调度，营造良好的产业发展环境。

加快推进基础设施体系建设。永武高速公路路基基本形成；龙厦铁路建设全面推进；建成市汽车客运中心主体工程。争取龙岩50万伏输变电工程、坑口火电厂二期和漳平电厂改扩建完成主体工程建设，力争永春至永定高速公路控制性工程开工建设。按照时速200公里、双线电气化标准设计建设赣龙铁路复线，确保年内立项，力争年内、确保明年开工建设。加快推进上杭蛟洋至城关国高连接线、漳州古雷港至武平（闽赣界）高速公路龙岩段、永安至漳州高速公路漳平段、南平至三明至龙岩铁路、漳平至泉州至肖厝铁路、长汀至永安铁路、福厦漳龙城际铁路、武夷山至建宁至龙岩铁路等项目的前期工作。

稳步推进城市体系建设。认真贯彻《城乡规划法》，完成中心城市总体规划修编，完善城市新区、城市组团规划和产业、交通等专项规划。加快推进中心城市商务、物流商贸、人居三大板块建设，重点抓好首期商务写字楼板块、五星级旅游饭店、国际商贸中心、闽西交易城二期、龙门物流中心和功能综合配套的住宅小区等项目建设。加快发展中心城市教育、文化、医疗等公共事业，完成博物馆和会展中心主体工程建设。认真组织实施74个重点城建项目，打通中心城市13条断头路，完善城市路网，改善公共交通，加强绿化美化。关闭中心城市6家水泥污染企业，加大龙津河污染治理力度，加快第二水源和垃圾焚烧发电厂项目建设。坚持市区一体、权责一致的原则，推进重心下移，强化属地管理，进一步理顺体制，健全城市管理长效机制。加强对县域经济发展的指导、支持和服务，促进中心城市、县域城市和小城镇协调发展。

扎实推进“两个带动”。深入推进项目带动，认真组织实施“三二一”项目滚动推进计划，形成源源不断的新增长点；着力抓好110个重点项目建设，逐个项目落实工作班子、落实保障措施、落实工程质量，确保完成年度投资146.9亿元，加快推进龙烟精品“七匹狼”卷烟生产线、龙工工程机械液压设备、龙工工程机械铸锻件、希尔斯高档电梯、紫金山金铜矿联合开采等产业龙头项目建设，争取上杭紫金年产20万吨铜冶炼、马坑铁矿新增500万吨/年采选工程、龙马中重型卡车等项目年内开工建设；高度重视项目策划、开发和储备工作，加大项目前期投入，促进项目生成落地；继续对项目工作实行严格的目标管理，强化领导挂钩责任制、分级管理制、业主负责制和督查联动机制。加快推进品牌带动，完善扶持品牌的政策体系，强化要素支持，健全推介机制，加强品牌保护；重点培育一批优势明显的产业产品品牌、技术领先的科技品牌、影响广泛的服务品牌和带动力强的区域品牌；加强产品质量安全工作，支持企业参与制订并采用国际、国家和行业标准，健全产品质量安全监管体系。

2、坚持好字优先，着力转变经济发展方式。按照好字当头求先行的要求，大力推动经济发展方式转变，努力实现速度和结构质量效益相统一，经济发展和人口资源环境相协调。

增强自主创新能力。完善和落实支持自主创新的政策措施，充分发挥企业作为技术创新主体的作用。支持企业创建技术中心，实施技术创新项目，着力突破制约经济社会发展的关键技术。加快市科技创业园建设，扎实推进国家环保除尘产品质量监督检验中心筹建工作。用好“6·18”项目成果交易会平台，加快建设面向重点产业的8个公共技术服务平台，鼓励企业与高等院校、科研机构共建研发机构和开展研发活动，加强知识产权保护。深入实施“人才强市”战略，统筹抓好以高层次人才和高技能人才为重点的各类人才队伍建设，创新人才工作体制机制，激发各类人才的创造活力和创造热情。

加快产业优化升级。在改造和提升传统产业的同时，大力发展高新技术产业和非资源型产业，加快发展光伏产业等潜力产业。抓好水泥产业的总量控制和布局调整，继续做好淘汰落后水泥产能工作。加大力度整治违规建设、审批手续不全的煤炭生产企业，加快推进联合改造，积极开发煤化工产品。认真做好对接国家西气东输二期工程项目前期工作。大力发展高岭土精深加工。推进关闭不符合国家产业政策的小钢铁、小铁合金企业和小火电机组。规范小水电建设和运行。加强地质找矿工作，推进能源资源的整合，提高能源资源的保护勘查开发利用水平。

加大节能减排和环境保护力度。强化节能减排工作，制订实施有利于节能减排的财税、价格等激励政策，加强监督管理，严格环保执法，突出抓好冶金、火电、化工、建材等高耗能行业和高耗能企业节能，减少污染物排放，全面推行清洁生产。精心组织开展“循环经济年”活动。做好全国第一次污染源普查工作，加大汀江、九龙江、闽江流域水环境综合整治力度，加强大气污染、垃圾、噪音等综合整治，推进县级污水处理厂、垃圾无害化处理场建设，控制农村面源污染，强化城乡饮用水源保护。坚持最严格的耕地保护制度，加强土地节约集约利用，充分利用荒地、坡地建设基础设施和发展工业项目，做好第二次全国土地调查工作；进一步整顿和规范矿产资源开发秩序，控制“青山挂白”和新的水土流失；抓好植树造林，加强护林防火。

3、坚持城乡统筹，着力推进新农村建设。始终把“三农”工作作为重中之重，按照“多予少取放活”的方针和形成城乡经济社会发展一体化新格局的要求，巩固完善强化支农惠农政策，在继续抓好“十镇百村”新农村建设试点的基础上，推进上杭古田、漳平永福新农村建设综合推进实验区和10个新农村示范村建设以及23个新农村建设村庄整理试点示范工作，努力促进农业农村发展和农民增收。

大力发展现代农业。毫不放松粮食生产，稳定粮食播种面积，加大对种粮农民扶持力度，按照依法自愿有偿原则推进土地承包经营权流转，遏制耕地抛荒，提高粮食综合生产能力和供给保障能力。大力推进农业标准化生产，精心组织实施“一村一品”产业发展示范工程，抓好生猪标准化生产工程、百村竹业工程和优质高山茶工程建设，积极发展畜牧水产、林竹、花卉等产业。加强农业科技创新和成果转化，强化农业技术推广服务。落实动物疫病和植物病虫害防控措施，提高农产品质量安全水平。坚持用工业的理念谋划农业发展，大力发展畜禽、林竹、果茶、蔬菜等的精深加工，做好清理整顿木材经营加工企业工作，努力培育农产品加工专业园区，推动农产品生产的规模化、标准化和工厂化，推进农业产业化经营。

加强农村基础设施建设。整合涉农部门项目建设资金，统筹安排好农村“六通”工程、水利“六千”工程、农业综合开发、烟田基础设施等项目建设。健全防灾减灾救灾体系，完成防汛预警系统二期工程建设，完善短时灾害性天气预报预警应急服务系统，配合做好组建森林武警部队工作。推进农村信息化工程建设，建成农村远程教育网络。强化村镇规划建设管理，抓好5个乡镇、300个村的村庄规划，做好10个乡镇、50个村的绿化试点工作，深入开展“家园清洁行动”。加大对革命基点村、少数民族乡村建设的扶持力度。

拓宽农民增收渠道。加快发展乡镇“三农”服务中心，加强农村“六大员”和基层农技队伍建设，充分发挥下派驻村干部服务“三农”的积极作用。认真贯彻《农民专业合作社法》，支持发展农民专业合作组织，健全农村社会化、专业化服务体系。坚持务农、务工、创业三种培训一起抓，强化农村富余劳动力转移就业培训，完成农村劳动力各类技能培训10万人，力争向“10＋3”产业推荐技术工人和熟练工人3.5万人。大力发展劳务经济。拓展农村市场，加快农村现代市场流通体系建设，搞活农产品流通。加大扶贫开发力度。

4、坚持改革开放，着力增强发展动力。以纪念改革开放30周年为契机，开展新一轮解放思想大讨论，把解放思想落实到项目带动和品牌带动上，促进加快重要领域和关键环节改革步伐，拓展对外开放的广度和深度，为推动科学发展提供体制保障和强大动力。

深化体制改革。全面推进农村改革，深化集体林权制度改革，完善林权抵押贷款等配套措施；推动乡镇机构改革，积极稳妥化解乡村债务；抓好支农信贷综合试点。推进国有企业改革，争取完成15家市属国有企业产权改革，完善现代企业制度。加快国有资产管理体制改革，实施国有资本收益收缴和经营预算制度，加强非经营性国有资产管理。深化财税体制改革，强化预算管理和监督，加快公共财政体系建设。继续抓好人事制度、投资体制和社会事业领域改革。鼓励、支持、引导非公有制经济发展，落实和完善相关政策措施，健全服务体系。鼓励支持企业改制上市，力争新组建3家股份有限公司，新增3家股份有限公司进入上市辅导期、3家企业上市。

提高开放水平。加大招商引资力度，重点开展对珠三角、长三角等国内产业集聚地区和港澳台、东南亚以及欧美、日韩、澳新等发达国家和地区的招商；加强驻点招商、以商引商、中介招商、专业招商和园区招商，提升参与和组织“5·18”、“9·8”、“11·18”等重大招商活动的层次和水平，增强招商引资实效；积极引进世界500强、行业龙头企业和高新技术企业，引导更多外资投向高新技术产业和先进制造业，加强现代服务业、基础设施和社会事业项目引进，提高利用外资质量。落实支持外经贸发展的政策措施，抓好大通关建设，壮大外贸经营主体，加快发展外贸服务中介机构，优化出口产品结构，扩大外贸出口总量。加强区域协作，大力推进与厦门的山海协作、厦泉漳龙城市联盟和闽西南五市、闽粤赣十三市交流合作。

密切对台交流合作。认真贯彻中央和省对台工作部署，大力推进对台经济文化交流合作。制订鼓励台商投资政策，积极承接台资企业转移，支持在岩台资企业发展。加强与台湾机械、纺织、电子、铜等制造业和物流、商贸、旅游等服务业的合作。加快推进漳平国家级台湾农民创业园建设。发挥闽西作为台胞重要祖籍地优势，以客家文化为纽带，加强客家族谱研究，积极申办“客家之声”广播频率并争取在台湾播出，继续做好申办客家语电视频道工作，筹办海峡两岸三地客家山歌、绝技邀请赛，推进文化交流和民间往来。

5、坚持以人为本，着力促进社会和谐。围绕着力民生、着力民心，顺应人民群众过上更好生活的新期待，多为群众办好事、办实事，推动和谐社会建设，做到发展成果由人民共享。

更加注重社会建设。坚持教育优先，加大教育投入，改善农村办学条件，稳步发展高中阶段教育，大力发展职业教育，提升高等教育质量，促进教育均衡、协调发展。健全农村三级医疗卫生服务网络，扩大农村新型合作医疗受益面，推进农村大病统筹医疗保险试点工作，加快发展城市社区卫生服务，抓好城乡医疗救助试点扩面工作，完善公共卫生服务、医疗服务和医疗保障体系。加强人口与计生工作，稳定低生育水平。坚持实施积极的就业政策，认真贯彻《就业促进法》和《劳动合同法》，确保新增城镇就业2.7万人，实现下岗失业人员再就业1.3万人。扩大社会保障覆盖面，完善城乡居民最低生活保障制度，稳步提高企业退休人员养老保险待遇和“革命五老”人员保障水平，制订实施被征地农民社会保障办法，在有条件的地方积极探索实行村（居）主干生活津贴；加快推进经济适用房和廉租房建设；健全社会救助体系，建成市福利中心一期，做好优抚安置工作，鼓励和支持慈善事业发展。加强市场和价格监管，遏制生产资料尤其是农业生产资料价格过快上涨，依法打击串通涨价、囤积居奇、哄抬物价等违法违规行为，及时完善和落实对低收入群众的补助办法，保证市场供应和价格基本稳定。深入开展“平安龙岩”创建活动，加强公安基层基础建设，完善社会治安防控体系，依法防范和打击违法犯罪活动，健全社会矛盾调解机制，做好信访工作，确保社会稳定。认真贯彻《突发事件应对法》，完善突发事件应急管理机制。强化安全生产管理和监督，加大源头治理力度，加强安全事故隐患排查和整治，健全重大危险源监控制度，有效遏制各类安全事故发生。加强社区建设，完善基层管理和服务网络。深入开展群众性精神文明创建活动，力争进入省级文明城市行列。巩固军政军民团结，提高“双拥”共建水平。加强国防动员、民兵预备役、人民防空建设，促进国防后备力量建设与经济协调发展。完成第二次全国经济普查工作任务。继续抓好人事、机构编制、统计、民族宗教、外事侨务、老区、库区移民、对口援藏、气象、地震、档案、地方志、科普、老龄、妇女儿童、残疾人等工作。

推动文化发展繁荣。加强公益性文化事业建设，加快完善社区和乡村文化设施，构建公共文化服务体系。大力发展文化产业，繁荣文化市场。积极发展闽西特色文化，着力打造红色文化、客家文化和河洛文化品牌。做好永定土楼申报世界文化遗产工作和冠豸山申报世界自然遗产前期工作，办好土楼文化节和龙岩市第六届旅游节，扎实推进古田会议会址群维修保护三期工程建设、培田古民居保护与开发、闽西革命历史博物馆改版和电视剧《客海往事》、《共和国摇篮》拍摄等工作。加强历史文化名城、名镇、名村和非物质文化遗产保护与开发，做好第三次全国文物普查工作。繁荣新闻出版、广播电视和文学艺术。深入开展“扫黄打非”等专项整治行动，加强网络文化建设与管理。广泛开展全民健身运动，办好第四届市农运会，积极支持办好北京2008年奥运会和残奥会。

着力办好惠民实事。城乡教育方面，免除4.4万城市义务教育阶段学生学杂费，免费为25.2万农村义务教育阶段学生提供教科书，补助9.5万农村义务教育阶段寄宿生生活费；完成第三期28.8万平方米农村中小学D级危房改造，建成新校舍30万平方米；开展“希望工程2008”系列助学公益活动。医疗卫生方面，选派百名医生帮扶百所乡镇卫生院，改善15所乡镇卫生院医疗设备，培养100名乡镇卫生院医疗技术骨干。文化建设方面，实施14所乡镇文化站改造完善工程；新增903个20户以上自然村通广播电视。社会保障方面，健全劳动力就业服务体系；实施以大病统筹为主的城镇居民基本医疗保险；实施中心城市保障性住房建设工程；实施“造福工程”搬迁1.05万人；提高农村五保户供养标准；实施残疾人康复工程。农村基础设施方面，继续实施土地整理；建设户用沼气池7800口；实施农村饮水、水土流失综合治理等农村水利建设六项工程。环境保护方面，关闭水泥污染企业27家、机立窑生产线31条，淘汰落后水泥产能379万吨；完成生态公益林管护机制改革。食品药品安全方面，加大力度治理“餐桌污染”，建设“食品放心工程”；实施农村药品供应网络和监督网络建设工程。“三农”服务方面，提高农村“六大员”津贴补助标准，实施“万村千乡”市场工程、农村现代化流通服务网络工程和农村电影放映工程。中心城市建设方面，实施邮政便民服务进社区工程；优化公交线路，加快场站建设，更新公交车辆60部。

三、进一步加强政府自身建设

深化行政管理体制改革，加强政府自身建设，着力转变职能、理顺关系、优化结构、提高效能，加快构建权责一致、分工合理、决策科学、执行顺畅、监督有力的行政管理体制，更好地推进科学发展、促进社会和谐，更好地体现对人民群众高度负责。

1、转变政府职能，建设服务政府。全面正确履行政府职能，在加强和改善经济调节、市场监管的同时，更加注重社会管理和公共服务，促进基本公共服务均等化。深化行政审批制度改革，减少和规范行政审批，减少政府对微观经济运行的干预。完成县级行政服务中心建设，构建市、县两级协调联动的行政服务体系。推进政府绩效评估，加强效能监察，精简会议和文件，进一步提高政府办事效率。推行电子政务，加强政府网站建设。加强公务员队伍建设，进一步提高为经济社会发展服务、为人民服务的能力和水平。

2、牢记为民宗旨，建设责任政府。坚持全心全意为人民服务，真诚倾听群众呼声，真实反映群众愿望，真情关心群众疾苦，千方百计为人民群众排忧解难。健全政府职责体系，完善行政问责制度，严肃法纪政纪，坚决改变有令不行、有禁不止的现象。把“两个带动”作为有效运作的重要抓手，精心组织实施，以项目带动集聚要素、落实工作、增强后劲，以品牌带动提升质量、增进效益、提高水平。围绕事关发展、牵动全局、影响民生的深层次矛盾和问题，用心、细心、耐心运作，创造性地开展工作，不断拓展作为空间。

3、推进依法行政，建设法治政府。加强民主法制建设，扩大人民民主，发展基层民主，拓宽民主渠道，健全民主制度。深入开展法制宣传教育，规范行政行为，完善行政执法责任制、执法过错责任追究制、评议考核制和行政赔偿制，加强行政复议和规范性文件合法性审查工作，促进各级政府、政府各部门和全体公务员严格按照法定权限和程序行使权力、履行职责。依法接受人大及其常委会的法律监督、工作监督，主动接受政协的民主监督，自觉接受社会各界和人民群众的监督，提高办理人大代表建议和政协提案的质量。坚持科学决策、民主决策、依法决策，规范行政决策行为。认真落实《政府信息公开条例》，完善各类公开办事制度，大力推行政务公开，健全与群众利益密切相关重大事项的决策公示、听证等制度，切实保障群众的知情权、参与权、表达权和监督权。

4、坚持从严治政，建设廉洁政府。把反腐倡廉建设放在更加突出的位置，旗帜鲜明地反对腐败。加强领导干部廉洁自律工作，牢记“两个务必”，提高拒腐防变能力。认真落实党风廉政建设责任制，在坚决惩治腐败的同时，更加注重治本、注重预防、注重制度建设，拓展从源头上防治腐败工作领域。加强廉政文化建设，加大监察、审计监督力度，严肃查处各类违法违纪案件。坚决纠正损害群众利益的不正之风，保证人民赋予的权力始终用来为人民谋利益。

各位代表，时代赋予重任，奋斗铸就辉煌。让我们紧密团结在以胡锦涛同志为总书记的党中央周围，在省委、省政府和市委的领导下，高举中国特色社会主义伟大旗帜，以邓小平理论和“三个代表”重要思想为指导，深入贯彻落实科学发展观，解放思想，开拓创新，奋力拼搏，乘势而上，在新的起点上推进龙岩又好又快发展，更好地发挥龙岩在海西纵深推进中的前锋作用，为把龙岩建设成为生态型经济枢纽和海西重要增长极而努力奋斗！
